# Platelet Adhesion and Degranulation Induce Pro-Survival and Pro-Angiogenic Signalling in Ovarian Cancer Cells

**DOI:** 10.1371/journal.pone.0026125

**Published:** 2011-10-12

**Authors:** Karl Egan, Darragh Crowley, Paul Smyth, Sharon O'Toole, Cathy Spillane, Cara Martin, Michael Gallagher, Aoife Canney, Lucy Norris, Niamh Conlon, Lynda McEvoy, Brendan Ffrench, Britta Stordal, Helen Keegan, Stephen Finn, Victoria McEneaney, Alex Laios, Jens Ducrée, Eimear Dunne, Leila Smith, Michael Berndt, Orla Sheils, Dermot Kenny, John O'Leary

**Affiliations:** 1 Molecular and Cellular Therapeutics, The Royal College of Surgeons in Ireland, Dublin, Ireland; 2 Department of Histopathology, Trinity College Dublin, Dublin, Ireland; 3 Department of Obstetrics and Gynaecology, Trinity College Dublin, Dublin, Ireland; 4 The Biomedical Diagnostics Institute, Dublin City University, Dublin, Ireland; 5 Fluidigm Corporation [Europe], Amsterdam, Netherlands; National Taiwan University Hospital, Taiwan

## Abstract

Thrombosis is common in ovarian cancer. However, the interaction of platelets with ovarian cancer cells has not been critically examined. To address this, we investigated platelet interactions in a range of ovarian cancer cell lines with different metastatic potentials [HIO-80, 59M, SK-OV-3, A2780, A2780cis]. Platelets adhered to ovarian cancer cells with the most significant adhesion to the 59M cell line. Ovarian cancer cells induced platelet activation [P-selectin expression] in a dose dependent manner, with the most significant activation seen in response to the 59M cell line. The platelet antagonists [cangrelor, MRS2179, and apyrase] inhibited 59M cell induced activation suggesting a P2Y12 and P2Y1 receptor mediated mechanism of platelet activation dependent on the release of ADP by 59M cells. A2780 and 59M cells potentiated PAR-1, PAR-4, and TxA2 receptor mediated platelet activation, but had no effect on ADP, epinephrine, or collagen induced activation. Analysis of gene expression changes in ovarian cancer cells following treatment with washed platelets or platelet releasate showed a subtle but valid upregulation of anti-apoptotic, anti-autophagy pro-angiogenic, pro-cell cycle and metabolic genes. Thus, ovarian cancer cells with different metastatic potential adhere and activate platelets differentially while both platelets and platelet releasate mediate pro-survival and pro-angiogenic signals in ovarian cancer cells.

## Introduction

Ovarian cancer is the fifth leading cause of cancer related deaths in women [1]. It is the most common gynaecologic malignancy and has the highest fatality to case ratio of all gynaecologic malignancies. The poor survival rate is the result of late stage diagnoses. Most patients are asymptomatic until the disease has metastasised [2]. Spread of ovarian cancer has been considered to occur primarily in the peritoneum [3]. However, autopsy and imaging studies [4], as well as evidence for the presence of micrometastases in the bone aspirates of early stage ovarian cancer patients [5] suggest that hematogenous metastasis is more common than previously thought.

During hematogenous dissemination, the ability of circulating tumour cells to interact with platelets is believed to promote their survival within the circulation and therefore facilitate metastasis. Pre-clinical animal experiments have demonstrated that pharmacologically [6] or genetically [7] induced thrombocytopenia, as well as defects in platelet function [7]–[10] are associated with reduced metastasis. The interaction of cancer cells with platelets is believed to confer a number of advantages that promote successful metastasis, including protection from immunological assault and evasion of immune surveillance [11], [12], the release of growth, angiogenic, and vascular permeability factors during activation and degranulation [13].

Thrombosis and thrombocytosis are frequent complications of ovarian cancer and are associated with poor prognosis [14]–[16], highlighting the importance of platelets in the pathology of ovarian cancer. However, the interaction between platelets and ovarian cancer cells has not been well studied. In this study, we aimed to characterise the interaction of platelets with ovarian cells, using a normal ovarian cell line [HIO-80] and ovarian cancer cells lines with different biological properties and metastatic potentials [59M, SK-OV-3, A2780, and A2780cis].

Firstly, we studied platelet adhesion to ovarian cancer cells under static conditions to determine if an adhesive interaction between platelets and ovarian cancer cells exists. Secondly, we assessed the ability of ovarian cancer cells to induce platelet activation and degranulation [P-selectin expression]. After establishing that platelets adhere to ovarian cancer cells, and ovarian cancer cells are capable of inducing platelet activation and degranulation, we next assessed gene expression changes at the transcriptome level in ovarian cancer cells treated with platelets or platelet releasate. Our results show differential interactions between platelets and ovarian cancer cell lines, not only in terms of platelet adhesion and activation, but also in gene expression changes in cancer cells treated with washed platelets or platelet releasate. Multiple interactions occur between platelets and ovarian cancer cells involving factors released by platelets and cancer cells, as well as direct platelet–ovarian cell interactions. This interaction results in a pro-survival, pro-angiogenic signal for the ovarian cancer cell.

## Methods

### Ethics statement

Blood collection for this study was approved by the Royal College of Surgeons in Ireland ethics committee and written informed consent was obtained from all donors prior to phlebotomy.

### Reagents

All reagents were purchased from Sigma-Aldrich [St Louis, MO, USA] unless otherwise indicated. Collagen [soluble calf skin], Adenosine-5′-Diphosphate, Epinephrine, and Arachidonic Acid were obtained from BioData [Horsham, PA, USA]. Alexa Fluor-488-labelled Phalloidin, Calcein AM, and fibrinogen were obtained from Invitrogen [Carlsbad, CA, USA]. Phycoerythrin [PE]-labelled anti human P-selectin [mouse IgG], PE-labelled mouse IgG isotype control, and PE-labelled anti human CD42a [mouse IgG] antibodies were purchased from BD Pharmingen [San Diego, CA, USA].

### Cell lines

A selection of ovarian cell lines of epithelial origin were chosen for inclusion in this study as epithelial ovarian cancers are the most common histological type. HIO-80 [a gift from the Fox Chase Cancer Center, Philadelphia, PA] represents a non-tumorigenic normal human ovarian epithelial cell line, which has been immortalised by transfection with a plasmid encoding for the SV40 large T gene. The HIO-80 cells were maintained in a 1∶1 mixture of medium 199 and MCDB-105 supplemented with 10% fetal bovine serum, 2 mM L-glutamine, 100 units/ml penicillin, 100 ug/ml streptomycin and 0.2 IU/ml of recombinant human insulin as recommended by Fox Chase. 59M [European collection of cell cultures (ECACC), Salisbury, UK] represents a tumorigenic human ovarian epithelial carcinoma of endometrioid type with clear cell components, originally isolated from ascites of a 65 year old patient with metastatic ovarian cancer. 59M cells were maintained in DMEM, 1 g glucose/L, supplemented with 10% fetal bovine serum, 2 mM L-glutamine, 100 units/ml penicillin, and 100 ug/ml streptomycin as recommended by ECACC. SK-OV-3 [American Type Culture Collection (ATCC), Manassas, VA, USA] represents a tumorigenic human ovarian epithelial adenocarcinoma, originally isolated from ascites of a 64 year old patient with metastatic ovarian cancer. SK-OV-3 was cultured with McCoy's 5A media, supplemented with 10% fetal bovine serum [Invitrogen, The Netherlands], 100 units/ml penicillin and 100 ug/ml streptomycin as recommended by ATCC. A2780 [ECACC, Salisbury, UK] is derived from an ovarian serous epithelial tumour tissue in an untreated patient. The cisplatin-resistant cell line A2780cis was developed by chronic exposure of the parent cisplatin-sensitive A2780 cell line to increasing concentrations of cisplatin. Both cell lines were maintained in RPMI 1640 medium supplemented with 10% fetal bovine serum, 2 mM L-glutamine, 100 units/ml penicillin and 100 ug/ml streptomycin as recommended by ECACC. All cell lines were grown in standard conditions in a humidified atmosphere containing 5% CO_2_ at 37°C.

### Platelet preparation

Blood was obtained from healthy donors who had not taken medications known to affect platelet function for at least 10 days. Blood was collected by venipuncture through a 19-gauge butterfly needle without a tourniquet, to avoid platelet activation. Platelets were prepared as previously described [17]. In brief, for the preparation of platelet-rich plasma [PRP], blood was collected into a syringe containing 3.2% trisodium citrate as anticoagulant [10% vol/vol, then centrifuged at 170 g for 10 minutes at room temperature. For the preparation of washed platelets, blood was collected into Acid-Citrate-Dextrose [ACD: 38 mM citric acid, 75 mM sodium citrate, 124 mM D-glucose] as anticoagulant [15% vol/vol]. Blood was centrifuged at 170 g for 10 minutes at room temperature. PRP was acidified to pH 6.5 with ACD, and PGE_1_ [1 μM] was added to avoid platelet activation during centrifugation. Platelets were pelleted by centrifugation at 720 g for 10 minutes. The supernatant was removed and the platelet pellet was resuspended in JNL buffer [130 mM NaCl, 10 mM sodium citrate, 9 mM NaHCO_3_, 6 mM D-glucose, and 0.9 mM MgCl_2_, 0.81 mM KH_2_PO_4_, and 10 mM Tris, pH 7.4] to a concentration of 2.5×10^8^/mL and supplemented with 1.8 mM CaCl_2_.

### Platelet adhesion assay

The platelet adhesion assay was performed as previously described [18]. In brief, ovarian cells were seeded in a 96 well plate [Nunc, Thermo Fisher Scientific, Braunschweig, Germany] at a concentration of 5×10^4^/ml and grown until confluent. Washed platelets [1.5×10^8^/ml] preloaded with Calcein AM [2 µg/ml] were allowed to adhere to the cells for 45 minutes at 37°C. The total fluorescence [485/535 nm] per well was measured using a Wallac 1420 Victor2™ multilabel counter [Perkin Elmer, Waltham, MA., USA] plate reader. The plate was washed three times, 100 ml of JNL was added to each well of the plate, and the remaining fluorescence was read. % Platelet adhesion was calculated as [remaining fluorescence – blank]/[total fluorescence – blank] ×100. Platelet adhesion to ovarian cells was imaged by fluorescence microscopy. When confluent, cells were detached with 7 mM EDTA in Dulbecco's PBS, washed twice, and resuspended in JNL supplemented with 1.8 mM CaCl_2_. Cells [1×10^5^/ml] were incubated on a BSA coated poly-L-lysine slide for 45 minutes at 37°C. Following adhesion, washed platelets [50×10^6^/µl] were added to the slide and incubated for 45 minutes at 37°C. Samples were fixed with 3.7% paraformaldehyde for 15 minutes at room temperature and permeabilised with 0.1% Triton X-100. Platelets and ovarian cells were stained with Alexa Fluor-488 Phalloidin [41.25 nM,] for 15 minutes at room temperature. Platelets were specifically stained with phycoerythrin labelled anti-human CD42a antibody [1.25 µg/ml] for 2 hours at 37°C. The slides were mounted and imaged by fluorescence microscopy.

### Platelet activation

The ability of ovarian cancer cells to induce platelet activation (P-selectin expression) was assessed by a flow cytometry based assay modified from Nylander et al [19]. In a total reaction volume of 100 μl, 10 μl of PRP was incubated with ovarian cancer cells [0 – 1.5×10^6^/ml,] in the presence of a PE-labelled anti human P-selectin antibody [1.25 μg/ml] or an appropriate isotype control [1.25 μg/ml]. All incubations were performed at room temperature for 15 minutes. The reaction was then terminated with 1 ml of JNL buffer prior to analysis by flow cytometry. Samples were analysed using a BD FACS Calibur [Becton Dickinson, Palo Alto, CA, USA] within 1 hour. The instrument was set to measure size [forward scatter, FSC], granularity [side scatter, SSC] and cell fluorescence. Using a log FSC vs. log SSC dot plot, a two dimensional analysis gate was drawn around the platelet population, and a fluorescence histogram [log FL2 vs. count] was obtained for 10000 platelet events for each sample. Data was analysed using CellQuest Pro software and expressed as percentage of platelets that were P-selectin positive relative to the isotype control.

### Isolation of platelet releasate

Washed platelets [2.5×10^8^/ml] were stimulated with both TRAP [Thrombin receptor activating peptide, 20 μM] and Collagen [190 μg/ml] and stirred in a BioData PAP-4 light transmission aggregometer [Horsham, PA, USA] at 37°C for 15 minutes. The platelet aggregate was centrifuged at 720 g for 10 minutes. The supernatant was then aspirated and filtered through a syringe filter with a 0.22 μm PVDF membrane to remove platelet microparticles.

### MTT assay

To optimise the concentration of platelet releasate for tumour cell exposure, a series of MTT cell proliferation assays [Roche Diagnostics Ltd, United Kingdom] were performed according to the manufacturer's instructions to determine the maximum concentration that could be applied to cells without negatively impacting on cell growth or survival. Cells were seeded into 96-well cell culture plates at 2×10^4^ cells/well and cultured for 24 hours to allow cell attachment. Once attached to the plates, growth media was aspirated and cells were briefly washed with 200 μl of pre warmed PBS. Cells were then exposed to serial dilutions of platelet releasate from 1∶10 to 1∶10,000 in full media, serum free media or JNL buffer to allow optimisation of concentration to be used in subsequent gene expression based studies.

### Washed platelet/platelet releasate exposure for gene expression analysis

The optimal concentration of platelet releasate [determined by MTT] was applied to the cell lines and the levels of apoptosis were compared against cells grown in full media only using a Roche Apodirect TUNEL/Propidium iodide kit. As the 1∶1000 dilution of platelet releasate in full growth media was observed to be the highest concentration of platelet releasate in full media that did not impact on cell growth/viability for all cell lines and was subsequently shown to result in no significant difference in apoptosis compared to cells grown in full media alone it was determined that it was suitable to proceed with this concentration. Given that the platelet releasate was prepared from identical washed platelet preparations, the 1∶1000 dilution of platelet releasate directly informs the use of a 1∶1000 dilution of washed platelets for comparative study. Optimal concentrations of platelet releasate and washed platelets [1∶1000] were suspended in full culture media and applied to cultured cells in 75 cm^2^ flasks in triplicate. Cells were exposed to releasate or washed platelets for 6 hours after which total RNA was extracted from cells. Transcriptome analysis was performed using Affymetrix Human Exon Arrays.

### RNA extraction

Cells were washed briefly in PBS, trypsinised and centrifuged to remove supernatant. RNA was extracted using RNeasy mini kit [Qiagen Ltd., West Sussex, UK] according to the manufacturer's protocol. RNA quantity was assessed using a Nano-Drop ND-1000 Spectrophotometer [Wilmington, USA] and quality by an Agilent Bioanalyser 2100 and RNA 6000 Nano microfluidic chip assay [Santa Clara, USA]. RNA was stored at −80°C.

### Affymetrix array

100 ng of total RNA extracted from control cells and cells exposed to washed platelets/platelet releasate was labelled using the Ambion WT Expression kit [Ambion/Life Technologies, Austin, TX, USA] including the labelling controls from the Affymetrix Gene Chip Poly-A RNA Control Kit. As suggested by Affymetrix/Ambion, at each step of the sample preparation protocol, progress was monitored using both the Agilent 2100 Bioanalyzer and the Nanodrop spectrophotometer. Quality control [QC] required assessment after the first cycle RNA cleanup, after the second cycle single-strand cDNA cleanup and following ssDNA fragmentation. Prepared fragmented ssDNA was hybridised to the Affymetrix Human Exon 1.0 ST Array at 45°C for 16 hours following Affymetrix protocols for their GeneChip WT Terminal Labeling, GeneChip Hybridization Control and GeneChip Hybridization, Wash, and Stain kits [Affymetrix, Santa Clara, USA]. Following hybridization, the chips were washed and stained using the Affymetrix GeneChip Fluidics Station with appropriate 64 format assay protocol. Following staining and washing steps the Affymetrix GeneChip Scanner 3000 and Affymetrix GeneChip Operating Software was used for the management and initial processing of the expression data. The data from 45 exon arrays was subject to array quality control performed using the Affymetrix Expression Console. All controls were within the parameters suggested by Affymetrix. Following successful quality control standards assessment, the chip data was then analysed in depth using Biotique Systems XRAY analysis software. Each cell line was examined under three different conditions; resting cells, cells exposed to platelet releasate, and cells exposed to washed platelets. Each cell line and condition was assayed in triplicate. The software was used to compare the two exposure cohorts against the resting control and genes exhibiting a positive or negative fold change of greater than 1.5 and a significance of p≤0.05 examined.

### Fluidigm validation

Gene expression was validated using Fluidigm's high throughput qPCR 48.48 dynamic array system. Genes displaying the most significant fold changes in addition to genes involved in biologically relevant pathways were selected for validation. TaqMan® real-time PCR expression assays were used in conjunction with Fluidigm's microfluidic Biomark system. Samples were analysed in triplicate and results were compared with Affymetrix expression data. RNA was reverse transcribed to single stranded cDNA using a High Capacity cDNA RT Kit [Applied Biosystems, CA, USA] in 100μl reactions. Reactions contained 10 μl of buffer [10×], 4 μl of deoxynucleotide triphosphate [25×], 10 μl of random primers [10×], 5 µl of multiscribe RT enzyme [50 U/μl], 21 μl of nuclease-free water and 50 μl of extracted total RNA [20 ng/µl]. Prior to Fluidigm, high throughput qPCR, a pre-amplification step was performed following Fluidigm protocols; 1 ml of TaqMan assay for each of the genes of interest identified through XRAY analysis and endogenous controls were pooled and made to a final volume of 100 ml in 1× TE Buffer, pH 8.0. 1.25 ml of each sample was added to 2.5 ml Preamp Master Mix [AB] and 1.25 ml pooled assay mix to give a 5 ml reaction volume. This mixture was then subject to 14 amplification cycles of 95°C, 15 seconds and 60°C, 4 minutes before being diluted 1∶5 with DNAse and RNase free H_2_0 to give a final volume of 25 ml of pre-amplified cDNA. Fluidigm data was analysed using Fluidigm Real-Time PCR Analysis software [ver3.02] to yield relative quantitation values calibrated to normal [HIO-80] cells. Fold changes returned from Affymetrix analysis were plotted against those calculated from Fluidigm data and the correlation between the values was calculated using Graph Pad Prism [ver 5.02].

### Epithelial mesenchymal transition [EMT] expression analysis in platelet cloaked/releasate treated cells

The process of EMT is critical in the metastatic cascade, by facilitating entry of cells into the vascular system. RNA was reverse transcribed to single stranded cDNA using a High Capacity cDNA RT Kit [Applied Biosystems, CA, USA] in 100 µl reactions as above. A combination of the primary players in the EMT process [20] and additional genes identified in our laboratory as integral to the EMT process [data not shown] were examined as part of the profiling for EMT in ovarian cancer cells: Akt-1, ALDH1 A1, CDH1, PIK3CA, SNAIL1, SNAIL 2, TWIST 1, VIMENTIN, ZEB1, ZEB 2, TLR 4, MyD88 using TaqMan primer and probes [Life Technologies/Applied Biosystems: gene expression assays] according to the manufacturers instructions. Expression was examined in platelet and platelet releasate treated cells and compared with resting ovarian cancer cells for all the cell lines [HIO-80, 59M, SK-OV-3, A2780 and A2780cis]. Results were plotted as a volcano plot of p value versus fold change. The accession assay numbers for the genes examined are shown below:

GAPDH - Hs99999905_m1

AKT1 - Hs00178289_m1

ALDH1A1 - Hs00167445_m1

PIK3CA - Hs00180679_m1

SNAIL1 - Hs00195591_m1

SNAIL2 - Hs00950344_m1

ZEB1 - Hs01566407_m1

ZEB2 - Hs00207691_m1

TWIST - Hs00361186_m1

VIMENTIN - Hs00958116_m1

CDH1 - Hs01023895_m1

MYD88 - Hs00182082_m1

TLR4 - Hs00152939_m1

### Statistics

Data were analysed using GraphPad Prism 5.0 software [GraphPad Software Inc., San Diego, CA, USA]. Results are expressed as mean ± standard error of the mean.

## Results

### Adhesion of platelets to ovarian cancer cells under static conditions

**Figure 1:**
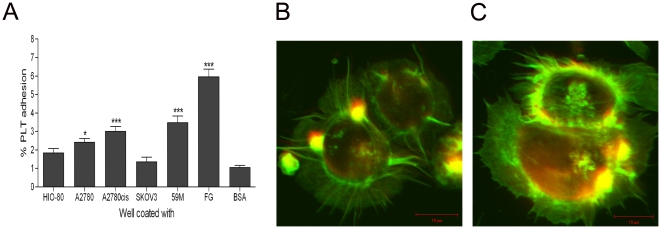
Platelet adhesion to a panel of ovarian cancer cell lines is heterogeneous under static conditions. [**A**] Platelet adhesion to ovarian cancer cells was quantified based on the fluorescence detection of labelled platelets. Platelet adhesion to fibrinogen and BSA were used as ± controls [n = 8 + SEM, *  =  p<0.05 vs. BSA]. Fluorescence microscopy images of platelets adhering to A2780 [**B**] and 59M [**C**] cells under static conditions [representative of n = 3]. Ovarian cancer cells and platelets were stained for actin [green], platelets were stained specifically for CD42a [red/yellow].

Platelet adhesion to ovarian cancer cell lines under static conditions was quantified based on the fluorescent detection of labelled platelets [Figure 1A]. Platelet adhesion to A2780 [2.4±0.2%, n = 8], A2780cis [3.0±0.2%, n = 8] and 59M [3.4±0.3%, n = 8] cells was significant compared to the negative control BSA [1.0±0.1%, n = 8]. Platelet adhesion to HIO-80 [1.8±0.2%, n = 8] and SK-OV-3 [1.3±0.2%, n = 8] cells was not significant compared to BSA. Adhesion to all 5 ovarian cancer cells was lower than to the positive control fibrinogen [5.9±0.4%, n = 8]. Fluorescence microscopy images clearly demonstrate platelet adhesion to the ovarian cancer cell lines A2780 and 59M [Figure 1B and C]. Consistent with the platelet adhesion assay, figure S1 demonstrates the minimal platelet adhesion to the HIO-80 cell line.

### Ovarian cancer cells induce platelet activation in a dose dependent manner

**Figure 2:**
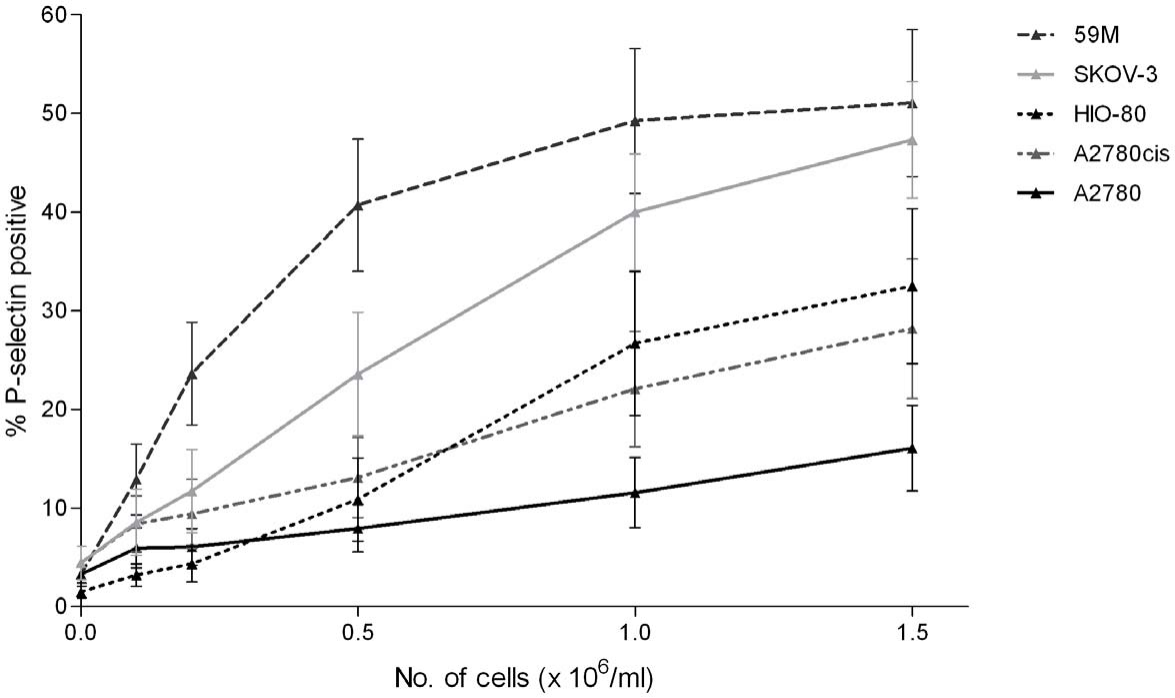
Ovarian cancer cell lines induce platelet activation in a dose dependent manner [n = 3–6, ± SEM]. Platelet activation [P-selectin expression] induced by a range of ovarian cell lines over a large concentration range [0.1–1.5×10^6^/ml] was measured by flow cytometry, based on platelet P-selectin surface expression. The most significant platelet activation was seen in response to the metastatic ovarian cancer cell lines SK-OV-3 and 59M.

The ability of ovarian cancer cells to induce platelet activation was quantified by flow cytometry. Increasing concentrations of ovarian cancer cells [0 – 1.5×10^6^ cells/ml] were added to PRP and platelet activation was assessed based on P-selectin expression. Ovarian cancer cells induced a dose-dependent increase in platelet activation [Figure 2]. The two metastatic ovarian cancer cell lines; SK-OV-3 [1.5×10^6^ cells/ml, 47±10.2% of platelets P-selectin positivity, n = 3] and 59M [1.5×10^6^ cells/ml, 51±18% P-selectin positivity n = 6] induced the most significant platelet activation. The lowest platelet activation was seen in response to the non-metastatic ovarian cancer cell line A2780 [1.5×10^6^ cells/ml, 16.1±5.2% P-selectin positivity, n = 6]. A2780cis [a cisplatin resistant daughter cell line to A2780] demonstrated higher platelet activation than its parent cell line [1.5×10^6^ cells/ml, 28.1±12.2% P-selectin positivity, n = 3]. The immortalised normal ovarian epithelial cells line HIO-80 also induced platelet activation [1.5×10^6^ cells/ml, 32.5±7.8% of platelets P-selectin positivity, n = 3], but to a lesser extent than 59M and SK-OV-3 cells.

### P2Y12/P2Y1 receptor and ADP/ATP antagonists inhibit platelet activation induced by 59M ovarian cancer cells

**Figure 3:**
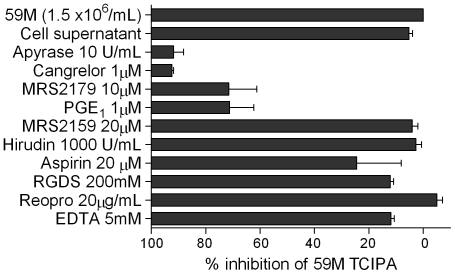
59M tumour cell induced platelet activation (TCIPA) is inhibited by cangrelor, MRS2179, and apyrase. This suggests a mechanism of platelet activation dependant on the platelet receptors P2Y12 and P2Y1, and ADP released by 59M cells into their supernatant. Other platelet antagonists such as hirudin, EDTA, abciximab, RGDS, and aspirin had no effect on 59M cell induced platelet activation.

Since 59M cells caused the most significant platelet activation, they were used to test the effect of a range of platelet inhibitors on ovarian cancer cell induced platelet activation. 59M ovarian cancer cells [1.5×10^6^ cells/ml, response normalised to 100% activation] were added to PRP pre-treated with inhibitors and platelet activation was measured by flow cytometry [P-selectin expression]. The concentrations of inhibitors were chosen based on preliminary flow cytometry and platelet aggregometry results that showed them to be antagonistic (data not shown). Antagonists against Thrombin [hirudin], integrin αIIbβ3 [Reopro and RGDS peptide], Cox-1 [aspirin], and calcium [EDTA], had no effect on 59M cell induced platelet activation [Figure 3]. Following treatment with the P2Y12 antagonist [cangrelor, 1 µM], the P2Y1 antagonist [MRS2179, 10 µM] or the ADP/ATPase (apyrase, 10 units/ml), platelet activation in the presence of 59M ovarian cancer cells was significantly diminished [1 μM Cangrelor - 92.4±0.64% inhibition, p<0.001; 10 µM MRS2179 – 71.4±10.52% inhibition, p = 0.01; 10 units/ml apyrase, 91.8±3.7% inhibition, p<0.001]. This suggests an ADP dependent mechanism of platelet activation by 59M cells, potentially mediated by the release of ADP by the cells into their supernatant [Figure 3].

### Ovarian cancer cells potentiate TRAP [thrombin receptor activating peptide], PAR 4 agonist, and arachidonic acid induced platelet activation in a dose dependent manner

**Figure 4:**
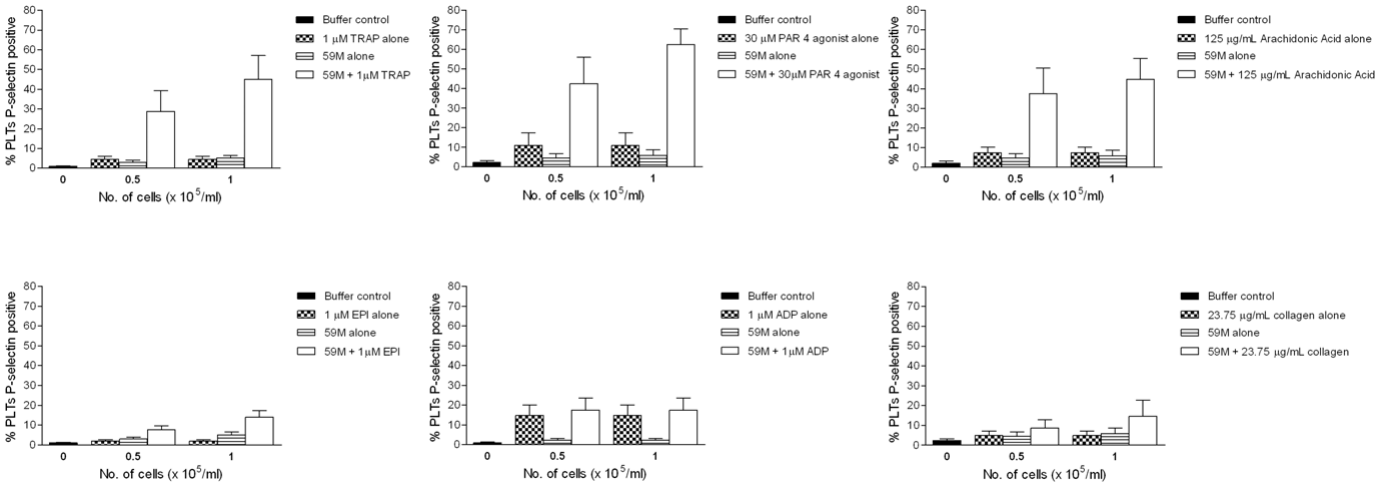
59M cells potentiate PAR-1, PAR-4, and TxA2 receptor mediated platelet activation. Activation occurs in a dose dependent manner [n = 3, + SEM]. At low concentrations, 59M [0.5–1×10^5^/ml], cells significantly potentiated TRAP, PAR4 agonist, and Arachidonic acid induced platelet activation [P-selectin expression]. This suggests a synergistic relationship between PAR-1, PAR-4, and TxA2 receptor mediated platelet activation and 59M induced platelet activation. Similar results are seen for A2780 cells [1–5×10^5^ cells/ml, data not shown]

Since thrombosis is a complex process that involves multiple agonists *in vivo*, we next asked if ovarian cancer cells modulated agonist [TRAP, PAR 4 agonist, Arachidonic Acid, ADP, epinephrine, and Collagen] induced platelet activation. To determine this, we assessed activation in platelets co-incubated with low ovarian cancer cell concentrations and low agonist concentrations. Platelet agonist concentrations that gave ≤20% platelet activation [P-selectin expression] were used. At low cellular concentrations [0.5–1×10^5^ cells/ml], 59M ovarian cancer cells potentiated PAR-1 [TRAP], PAR-4 [PAR4 agonist] and TxA2 receptor [Arachidonic Acid] mediated platelet activation [P-selectin expression], but had no effect on ADP, epinephrine, or collagen induced activation [Figure 4]. For example, in response to 1 μM TRAP and 1×10^5^ 59M cells/ml, platelet activation was 44±12.16% P-selectin positivity [n = 3, Figure 3] compared to 4.4±1.6% P-selectin positivity [1×10^5^ 59M cells/ml alone, n = 3] and 5.1±1.3% P-selectin positivity [1 µM TRAP alone, n = 3]. Conversely, in response to 1 µM ADP and 1×10^5^ 59M cells/ml, platelet activation was 17.3±6.2% P-selectin positivity [n = 3, Figure 4] compared to 2.4±1.4% [1×10^5^ 59M cells/ml alone, n = 3] and 14.73±5.3% [1 uM ADP alone, n = 3]. Similar results are also seen in response to A2780 cells [1 – 5×10^5^ cells/ml], but higher A2780 cell concentrations were required to induce a similar effect to 59M cells [data not shown]. The data suggests a synergistic relationship between PAR-1, PAR4, and TxA2 receptor mediated platelet activation and A2780/59M induced platelet activation. This synergistic interaction is also inhibited by the P2Y12 antagonist cangrelor [data not shown].

### Expression array analysis of ovarian cancer cells treated with washed platelets or platelet releasate using Affymetrix exon arrays

All arrays passed QC using Affymetrix QC software. Biotiques X-Ray software plug-in for Microsoft Excel was used to interrogate gene expression changes between treatments and cell types [Fold change >1.5 and p<0.05]. Analytical stringency was relaxed to fold changes of >1.5 to accommodate subtle but meaningful biological variation [p<0.05] that was contingent upon treatment. Tables 1, 2, 3, 4 depict the gene expression variations observed in cells treated with platelet releasate/washed platelets.

**Table 1:** Expression data for HIO-80 cells.

			Affymetrix		Fluidigm	
Cell Line	Exposure	Gene	Fold Change	Significance (*p* = )	Fold Change	Significance (*p* = )
HIO-80	Platelet Releasate	**LRP8**	1.50	0.0275	1.612	0.0000*
	Washed Platelet	**SERPINB2**	1.76	0.0016	1.6909	0.0000*
		IDI1	1.70	0.0218		
		PMM2	1.60	0.0188		
		**PCGF6**	1.59	0.0372	1.137	0.0077
		CCDC68	1.56	0.0245		
		**ZNF267**	1.55	0.0318	1.1571	0.0000*

Data presented according to whether cells directly exposed to platelets or releasate. Genes highlighted in bold were selected for validation. Fluidigm validation fold change and t-test *p* values indicated on right.

* *p*<0.00001.

**Table 2:** Expression data for 59M cells.

			Affymetrix		Fluidigm	
Cell Line	Exposure	Gene	Fold Change	Significance (*p* = )	Fold Change	Significance (*p* = )
59M	Platelet Releasate	**CCL2**	1.94	0.0137	2.83	0.0000*
		**TRAF1**	1.92	0.0013	3.50	0.0000*
		**HBEGF**	1.67	0.0034	2.01	0.0000*
		**CD274**	1.64	0.0487	2.09	0.0000*
		ANKRD1	1.60	0.0341		
		**TNFAIP2**	1.59	0.0018	2.51	0.0000*
		**BIRC3**	1.58	0.0303	1.88	0.0000*
		ICAM1	1.57	0.0012		
		IRAK2	1.56	0.0050		
		**SH2B3**	1.53	0.0060	2.20	0.0000*
		CSF2	1.52	0.0188		
		GCH1	1.52	0.0211		
		HIVEP1	1.51	0.0212		
	Washed Platelet	**TRAF1**	1.90	0.0056	2.87	0.0000*
		**CCL2**	1.78	0.0281	2.67	0.0000*
		**TNFAIP2**	1.63	0.0048	1.86	0.0000*
		**PDGFB**	1.50	0.0397	2.45	0.0000*
		**DSN1**	-1.60	0.0350	-1.09	0.0036

Data presented according to whether cells directly exposed to platelets or releasate. Genes highlighted in bold were selected for validation. Fluidigm validation fold change and t-test *p* values indicated on right.

* *p*<0.00001.

**Table 3:** Expression data for SK-OV-3 cells.

			Affymetrix		Fluidigm	
Cell Line	Exposure	Gene	Fold Change	Significance (*p* = )	Fold Change	Significance (*p* = )
SK-OV-3	Platelet Releasate	**CRLS1**	1.91	0.0346	-1.09	0.0338
		UBLCP1	1.82	0.0256		
		RPL7A	1.72	0.0290		
		AK3	1.66	0.0432		
		MGC71993	1.66	0.0299		
		**ANXA2**	1.64	0.0225	1.04	0.3191
		**KIAA1324**	1.58	0.0417	-1.05	0.1362
		PCNP	1.52	0.0455		
		SLC9A8	1.52	0.0480		
		TPM4	1.51	0.0356		
		ACTL6A	1.51	0.0380		
		C14ORF153	1.50	0.0190		
		**MUS81**	1.50	0.0222	-1.09	0.0809
		**CRIPAK**	-1.55	0.0105	-1.23	0.0007
		**ISOC2**	-1.69	0.0036	-1.09	0.0000*
		**VPS26B**	-1.69	0.0402	-1.03	0.5467
	Washed Platelet	**ASTN1**	1.58	0.0030	-1.03	0.9001

Data presented according to whether cells directly exposed to platelets or releasate. Genes highlighted in bold were selected for validation. Fluidigm validation fold change and t-test *p* values indicated on right.

* *p*<0.00001.

**Table 4:** Expression data for A2780cis cells.

			Affymetrix		Fluidigm	
Cell Line	Exposure	Gene	Fold Change	Significance (*p* = )	Fold Change	Significance (*p* = )
A2780cis	Washed Platelet	**KLK1**	1.63	0.0239	1.44	0.0309
		**ITGB2**	1.52	0.0497	1.23	0.0000*
		ABCD3	-1.50	0.0431		
		RMND1	-1.50	0.0335		
		ARL15	-1.50	0.0137		
		RAD18	-1.51	0.0327		
		CAPZA1	-1.51	0.0324		
		SPAST	-1.52	0.0200		
		C11ORF54	-1.53	0.0316		
		MAD2L1	-1.53	0.0407		
		MRPL33	-1.53	0.0328		
		RNASEH2B	-1.53	0.0384		
		DAB1	-1.54	0.0204		
		SMCHD1	-1.54	0.0359		
		DPY19L4	-1.56	0.0383		
		EIF2B3	-1.56	0.0310		
		C1ORF112	-1.56	0.0305		
		ZNF271	-1.56	0.0131		
		VGLL3	-1.56	0.0115		
		NDUFAF1	-1.58	0.0443		
		LARP7	-1.58	0.0400		
		SNRPA1	-1.59	0.0276		
		MED10	-1.60	0.0114		
		PTBP2	-1.60	0.0337		
		**STK17B**	-1.61	0.0304	1.13	0.0019
		PRIM2A	-1.62	0.0464		
		**GMNN**	-1.67	0.0259	1.02	0.4974
		ZNF706	-1.68	0.0259		
		CCDC76	-1.70	0.0263		
		RP9	-1.71	0.0387		
		**NAP1L4**	-1.81	0.0082	-1.19	0.0054

Data presented according to whether cells directly exposed to platelets or releasate. Genes highlighted in bold were selected for validation. Fluidigm validation fold change and t-test *p* values indicated on right.

* *p*<0.00001

In HIO-80 cells, following treatment with washed platelets or platelet releasate, seven genes were significantly up-regulated, with the greatest difference seen in response to washed platelets. The upregulated genes encode for proteins associated with ovarian cancer metastasis [SERPINB2/PAI2], metabolic activities [IDI1, PMM2] and gene expression/transcription [PCGF6, ZNF267].

59M cells also exhibited gene expression changes following treatment with platelet releasate and washed platelets. The biological processes affected involved anti-autophagy, anti-apoptotic and pro-angiogenic signalling pathways [TRAF2, CCL2, TNFAIP2, PDGFb]. Pro-proliferative [HBEGF, CSF2/GMCSF, IRAK2] immune suppression [CD274/PDL1] anti-apoptotic [BIRC3/CIAP], cell adhesion and migration [ICAM1] signalling was also altered in 59M cells treated with platelet releasate.

SK-OV-3 cells exhibited a greater difference in gene expression following treatment with platelet releasate compared to treatment with washed platelets. The largest fold change was observed for the gene encoding for Cardiolipin Synthase [CRLS1] responsible for cardiolipin [CL] production.

The A2780 cell line and its cisplatin resistant daughter cell line A2780cis used here as a model for recurrent chemo-resistant disease returned different responses to treatment with washed platelets or platelet releasate. The A2780 cell line did not show significant alteration of gene expression in response to either treatment. Conversely, the A2780cis cell line revealed a panel of genes up and down regulated following treatment with washed platelets but none following treatment with platelet releasate. Increased expression was observed in genes for cancer associated proteases [KLK1], cell adhesion/migration molecules [ITGB2/LFA-1], and reduced expression of genes involved in maintaining genomic instability [GMNN], inhibition of gene transcription/expression [CCDC7B, ZNF271, ZNF706, LARP7, RNASEH2B], pro-apoptotic regulation [STK17B/DRAK2] and immune response/evasion [CD58/LFA3].

### Validation of gene expression signature by the Fluidigm 48×48 dynamic array

**Figure 5:**
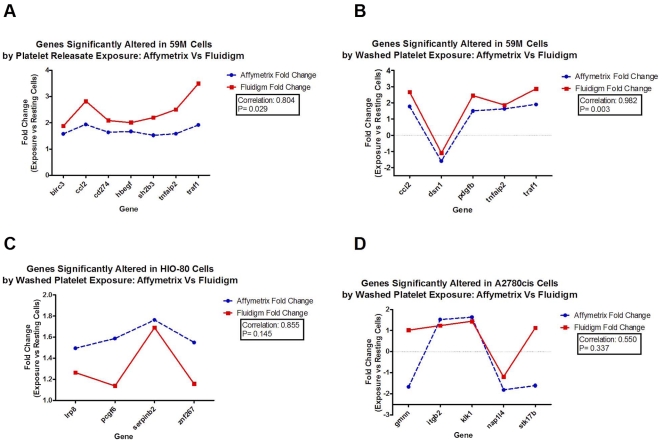
Fluidigm Dynamic Array Validation of significantly altered gene expression [fold change >1.5X; <1.5X; p<0.05]. This figure displays some examples of the correlation between the affymetrix array data and the fluidigm validation data. Correlation coefficients are displayed in the legend.

**Figure 6:**
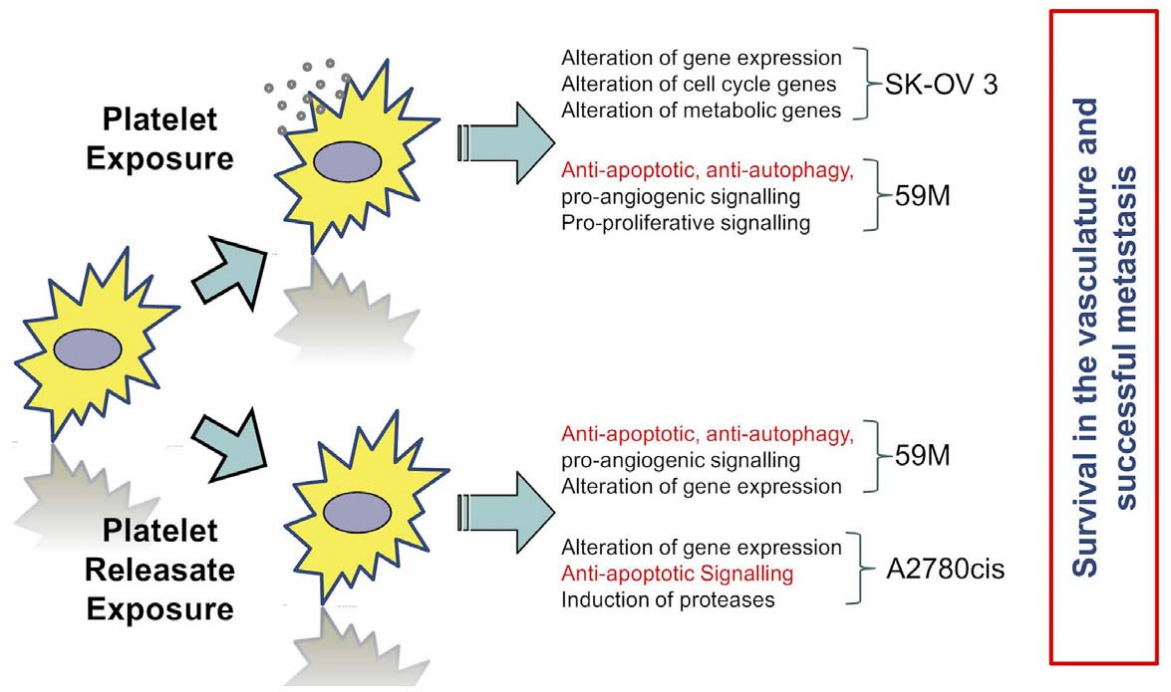
Summary analysis of gene expression changes due to platelets or platelet releasate. Ovarian cancer cells exposed to platelets and platelet releasate exhibit a variety of gene expression changes summarised in this figure.

Gene expression array results were validated using Fluidigm high throughput qPCR technology. Twenty five genes were selected along with two endogenous controls for analysis using Fluidigm's 48×48 Dynamic Array. The genes selected and the cell lines and conditions in which significant alteration of expression were observed by Affymetrix analyses are highlighted in bold in Tables 1, 2, 3, 4 below. Fold change values for samples revealed close correlation between Affymetrix and Fluidigm data sets for those genes observed to have undergone altered expression in the 59M, SK-OV-3 and A2780cis cell lines [Figure 5A, B, C, D]. Discrepancies between expression calculated by Affymetrix and TaqMan analyses are likely due to normalisation and summarisation methods. The expression score for each probeset for the arrays is derived using the standard Robust Multiarray Averaging [RMA] normalisation and summarisation methods. The RMA algorithm was used because compared to other approaches, it increases sensitivity to small changes between experiment and control samples and minimses variance across dymanic range, but in doing so it does compress calculated fold change values. This means generally the fold change value obtained with the affmetrixy array is smaller than that obtained with the TaqMan assay. The correlation for genes observed to undergo significant alteration of expression in both the normal [immortalised] HI0-80 and malignant 59M ovarian carcinoma cell lines was high [>0.8] and was statistically significant [p<0.05]. Figure 6 summarises the findings of the gene expression analysis.

### Expression analysis of EMT associated genes in ovarian cancer cells exposed to platelet releasate

**Figure 7:**
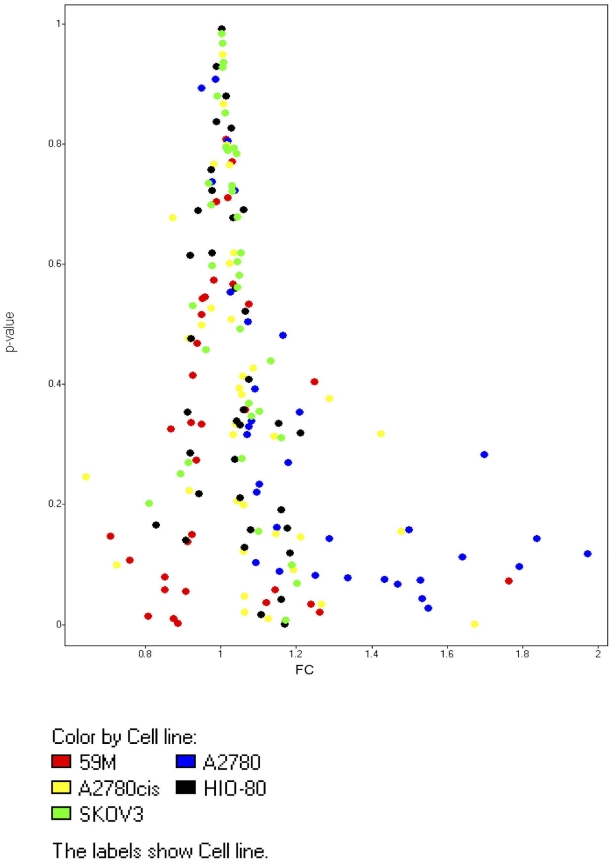
Volcano plot of TaqMan derived fold changes in EMT associated genes in cloaked ovarian cells. This figure displays fold change difference in relation to p value for EMT genes assessed in the cell lines.

EMT profiling was performed to see if interaction with platelets would induce EMT. Expression analysis of EMT associated genes demonstrated constitutive expression of the majority of EMT associated genes [Figure 7]. Importantly, platelet releasate did not induce/suppress EMT.

## Discussion

The results of the present investigation demonstrate a differential interaction between platelets and ovarian cancer cell lines, not only in terms of the effect of ovarian cancer cells on platelets, but also in the effect of platelets on ovarian cancer cells. Firstly, we used a panel of ovarian cell lines to assess platelet adhesion under static conditions. Adhesion across the 5 cells lines was extremely heterogeneous with significant platelet adhesion to the 59M, A2780, and A2780cis cell lines, while adhesion to the HI0-80 and SK-OV-3 cell lines was not significant compared to the BSA negative control [Figure 1]. The adhesion of platelets to all ovarian cancer cell lines was significantly less than to fibrinogen, suggesting that the adhesion is mediated by either a lower copy number receptor-ligand interaction, or a lower affinity interaction than platelet integrin αIIbβ3 mediated adhesion to fibrinogen. The molecular mechanisms mediating platelet adhesion to ovarian cancer cells remains to be elucidated. Secondly, we assessed the ability of ovarian cancer cells to induce platelet activation. We found that ovarian cancer cells induce platelet activation and degranulation in a dose dependent manner with the most significant platelet activation seen in response to the 59M and SK-OV-3 ovarian cancer cell lines [Figure 2].

Using 59M cells we then investigated the effect of different platelet inhibitors to determine the mechanism of ovarian cancer cell induced platelet activation. Following treatment with cangrelor [P2Y12 antagonist], MRS2179 [P2Y1 antagonist], or apyrase [ADP/ATPase], platelet activation in the presence of 59M ovarian cancer cells was greatly diminished, suggesting a P2Y12/P2Y1 dependent mechanism of activation, mediated by the release of ADP by 59M cells, as the 59M cell supernatant induced comparable platelet activation [Figure 3]. Consistent with the literature, other studies have also demonstrated ADP dependent platelet aggregation induced by cancer cells [21]. Of note, Uluckan et al have shown that treatment with APT102 [an ADPase] and aspirin in combination, inhibited B16 melanoma cell induced aggregation, and decreased metastasis in a murine B16 melanoma model of bone metastasis [22]. Italiano et al have previously shown that platelets differentially package angiogenic regulatory proteins in separate alpha granules that are subject to differential release depending on the platelet agonist [23]. Interestingly, Bambace et al have demonstrated that ADP induced platelet activation causes the release of VEGF [pro-angiogenic] but not endostatin [anti-angiogenic] *in vitro* [24], indicating a potential mechanism by which ovarian cancer cells could induce the release of pro-angiogenic but not anti-angiogenic factors by platelets *in vivo*.

Ovarian cancer cells alone induce activation of platelets. However, thrombosis is a complex process that involves many different agonists *in vivo*. Therefore, we examined how ovarian cancer cells modulate agonist induced platelet activation. At low cellular concentrations, both A2780 and 59M cells potentiate TRAP, PAR-4 agonist, and arachidonic acid induced platelet activation, but not ADP, epinephrine, or collagen induced platelet activation [Figure 4]. This suggests a synergistic relationship between PAR-1/PAR-4/TxA2 receptor mediated activation and the mode of ovarian cancer cell induced activation. Thrombin is the physiological ligand for the PAR-1 and PAR-4 receptors, and its generation is critical for coagulation. The expression of thrombin has been identified in ovarian cancer tissue [25] and is known to potentiate invasion in an *in vitro* model of ovarian cancer [26]. Holmes et al have also shown that TRAP treated platelets increase ovarian cancer cell invasion [27]. Prostaglandin synthesis is also increased in ovarian cancer, with a trend towards higher fold increases in pro-aggregatory TxA2 levels compared to anti-aggregatory PGI_2_ levels [28].

Having established that ovarian cancer cells interact with platelets, we next assessed the effect of platelet adhesion and platelet granule release on ovarian cancer cells. Overall analysis of gene expression changes in ovarian cancer cells following treatment with washed platelets or platelet releasate showed an upregulation of anti-apoptotic, anti-autophagy, pro-angiogenic, pro-cell cycle and metabolic genes in the treated ovarian cancer cells [Tables 1, 2, 3, 4]. However, ovarian cancer cells of variable phenotype showed differential gene expression profiles, possibly related to their underlying biology.

In HIO-80 cells, there was significant upregulation of genes encoding for proteins associated with ovarian cancer metastasis [SERPINB2/PAI2], metabolic activities [IDI1, PMM2] and gene expression/transcription [PCGF6, ZNF267] [29]. LRP8 [ApoER2] which showed significantly increased expression in response to platelet releasate exposure was also observed to be up regulated in response to washed platelet exposure and its activation has been observed to alter migratory capability in a non-tumorigenic breast epithelial [MCF 10A] cell model [30]. It also has a key role in mediating increased platelet activation and adhesion in association with other glycoproteins or clotting factors [31]–[34] and may promote signalling in cells via ApoER2. HIO-80 are a non-tumorigenic normal human ovarian surface epithelial cell line, which has been immortalised by transfection with a plasmid encoding for the SV40 large T gene. SERPINB2/PAI2 was the most significantly upregulated gene on validation and this upregulation may be explained by the fact that platelets can release TNFα [35] which in turn has been shown to induce matrix proteolytic enzyme production and basement remodelling by human ovarian surface epithelial cells providing a molecular mechanism linking ovulation and ovarian cancer risk [36]

59M cells exhibited aberrant upregulation of genes involved in anti-autophagy, anti-apoptotic and pro-angiogenic signalling [TRAF2, CCL2, TNFAIP2] [37]–[39]. Among the other pathways showing altered expression were pro-angiogenic signalling through increased PDGFb transcription in response to washed platelet exposure with further pro-proliferative [HBEGF, CSF2/GMCSF, IRAK2] immune suppression [CD274/PDL1] anti-apoptotic [BIRC3/CIAP], cell adhesion & migration [ICAM1] signalling [40]–[44].

SK-OV-3 cells exhibited significant overexpression in the Cardiolipin Synthase [CRLS1] gene, which is responsible for cardiolipin [CL] production. Anti-cardiolipin antibodies are associated with both solid and non-solid tumours and are associated with increased thrombocytosis [45]. This gene product [CRLS1] may also have an anti-apoptotic role as reduced CL expression is associated with increased apoptosis [46]. Genes associated with increased cell invasion/motility in breast and pancreatic cancers [ANXA2] were also dysregulated. Following treatment with platelet releasate, SK-OV-3 cells also showed expression changes in genes involved in, anti-autophagy in endometrial and ovarian cancer [KIAA1324/EIG121], as well as transcriptional regulation [ACTL6A], cell cycle[MUS81], cytoskeletal [TPM4] and homeostatic [TPM4] processes [47], [48].

The A2780 cell line and its cisplatin resistant daughter cell line A2780cis displayed different responses to the exposure treatments. The A2780 cell line did not show significant alteration of gene expression following treatment with either platelet releasate or washed platelets. Conversely, the A2780cis cell line revealed a panel of dysregulated genes following treatment with washed platelet but none following treatment with platelet releasate. Increased expression was observed in genes for cancer associated proteases [KLK1],cell adhesion/migration molecules [ITGB2/LFA-1], and reduced expression of genes involved in maintaining genomic instability [GMNN], inhibition of gene transcription/expression [CCDC7B, ZNF271, ZNF706, LARP7, MASEH2B], in pro-apoptotic regulators [STK17B/DRAK2] and immune response/evasion [CD58/LFA3] [49], [50].

TaqMan expression analysis of EMT associated genes demonstrated constitutive expression of the majority of EMT associated genes [Figure 7]. It appears that EMT related genes are effectively ‘primed’ and that interaction with platelets does not significantly alter the capacity of cancer cells to mount this type of response. Other studies have reported similar findings in breast cancer [51], [52].

In summary, our data shows for the first time that there is a potent dynamic interaction between ovarian cancer cells and platelets *in vitro*. This interaction involves platelet adhesion, platelet activation and degranulation and a resultant pro-survival and pro-angiogenic signal for the ovarian cancer cell that could potentially promote ovarian cancer cell metastasis. Further work is required to determine the significance of this interaction *in vivo.*

## Supporting Information

Figure S1 **Platelet adhesion to HIO-80 cells is minimal.** Fluorescence microscopy image demonstrating that platelet adhesion to HIO-80 cells is minimal. HIO-80 cells and platelets were stained for actin [green], platelets were stained specifically for CD42a [red].
(TIF)

## References

[1] Landen CN, Birrer MJ, Sood AK (2008). Early events in the pathogenesis of epithelial ovarian cancer.. J Clin Oncol.

[2] Holschneider CH, Berek JS (2000). Ovarian cancer: epidemiology, biology, and prognostic factors.. Semin Surg Oncol.

[3] Naora H, Montell DJ (2005). Ovarian cancer metastasis: integrating insights from disparate model organisms.. Nat Rev Cancer.

[4] Dvoretsky PM, Richards KA, Angel C, Rabinowitz L, Stoler MH (1988). Distribution of disease at autopsy in 100 women with ovarian cancer.. Hum Pathol.

[5] Braun S, Schindlbeck C, Hepp F, Janni W, Kentenich C (2001). Occult tumor cells in bone marrow of patients with locoregionally restricted ovarian cancer predict early distant metastatic relapse.. J Clin Oncol.

[6] Gasic GJ, Gasic TB, Stewart CC (1968). Antimetastatic effects associated with platelet reduction.. Proc Natl Acad Sci U S A.

[7] Camerer E, Qazi AA, Duong DN, Cornelissen I, Advincula R (2004). Platelets, protease-activated receptors, and fibrinogen in hematogenous metastasis.. Blood.

[8] Jain S, Russell S, Ware J (2009). Platelet glycoprotein VI facilitates experimental lung metastasis in syngenic mouse models.. J Thromb Haemost.

[9] Jain S, Zuka M, Liu J, Russell S, Dent J (2007). Platelet glycoprotein Ib alpha supports experimental lung metastasis.. Proc Natl Acad Sci U S A.

[10] Kim YJ, Borsig L, Varki NM, Varki A (1998). P-selectin deficiency attenuates tumor growth and metastasis.. Proc Natl Acad Sci U S A.

[11] Philippe C, Philippe B, Fouqueray B, Perez J, Lebret M (1993). Protection from tumor necrosis factor-mediated cytolysis by platelets.. Am J Pathol.

[12] Nieswandt B, Hafner M, Echtenacher B, Männel DN (1999). Lysis of tumor cells by natural killer cells in mice is impeded by platelets.. Cancer Res.

[13] Maloney JP, Silliman CC, Ambruso DR, Wang J, Tuder RM (1998). In vitro release of vascular endothelial growth factor during platelet aggregation.. Am J Physiol.

[14] Gungor T, Kanat-Pektas M, Sucak A, Mollamahmutoglu L (2009). The role of thrombocytosis in prognostic evaluation of epithelial ovarian tumors.. Arch Gynecol Obstet.

[15] Iodice S, Gandini S, Löhr M, Lowenfels AB, Maisonneuve P (2008). Venous thromboembolic events and organ-specific occult cancers: a review and meta-analysis.. J Thromb Haemost.

[16] Levitan N, Dowlati A, Remick SC, Tahsildar HI, Sivinski LD (1999). Rates of initial and recurrent thromboembolic disease among patients with malignancy versus those without malignancy. Risk analysis using Medicare claims data.. Medicine [Baltimore].

[17] Edwards RJ, Moran N, Devocelle M, Kiernan A, Meade G (2007). Bioinformatic discovery of novel bioactive peptides.. Nat Chem Biol.

[18] Stevens JM (2004). Platelet adhesion assays performed under static conditions.. Methods Mol Biol.

[19] Nylander S, Mattsson C, Ramström S, Lindahl TL (2004). Synergistic action between inhibition of P2Y12/P2Y1 and P2Y12/thrombin in ADP- and thrombin-induced human platelet activation.. Br J Pharmacol.

[20] Thomson S, Petti F, Sujka-Kwok I, Mercado P, Bean J (2011). A systems view of epithelial-mesenchymal transition signaling states.. Clin Exp Metastasis.

[21] Medina C, Jurasz P, Santos-Martinez MJ, Jeong SS, Mitsky T (2006). Platelet aggregation-induced by caco-2 cells: regulation by matrix metalloproteinase-2 and adenosine diphosphate.. J Pharmacol Exp Ther.

[22] Uluçkan O, Eagleton MC, Floyd DH, Morgan EA, Hirbe AC (2008). APT102, a novel adpase, cooperates with aspirin to disrupt bone metastasis in mice.. J Cell Biochem.

[23] Italiano JE, Richardson JL, Patel-Hett S, Battinelli E, Zaslavsky A (2008). Angiogenesis is regulated by a novel mechanism: pro- and antiangiogenic proteins are organized into separate platelet alpha granules and differentially released.. Blood.

[24] Bambace NM, Levis JE, Holmes CE (2010). The effect of P2Y-mediated platelet activation on the release of VEGF and endostatin from platelets.. Platelets.

[25] Yousef GM, Polymeris ME, Yacoub GM, Scorilas A, Soosaipillai A (2003). Parallel overexpression of seven kallikrein genes in ovarian cancer.. Cancer Res.

[26] Zhang T, Ma Z, Wang R, Wang Y, Wang S (2010). Thrombin facilitates invasion of ovarian cancer along peritoneum by inducing monocyte differentiation toward tumor-associated macrophage-like cells.. Cancer Immunol Immunother.

[27] Holmes CE, Levis JE, Ornstein DL (2009). Activated platelets enhance ovarian cancer cell invasion in a cellular model of metastasis.. Clin Exp Metastasis.

[28] Aitokallio-Tallberg AM, Viinikka LU, Ylikorkala RO (1988). Increased synthesis of prostacyclin and thromboxane in human ovarian malignancy.. Cancer Res.

[29] Croucher DR, Saunders DN, Lobov S, Ranson M (2008). Revisiting the biological roles of PAI2 (SERPINB2) in cancer.. Nat Rev Cancer.

[30] Minami SS, Sung YM, Dumanis SB, Chi SH, Burns MP (2010). The cytoplasmic adaptor protein X11alpha and extracellular matrix protein Reelin regulate ApoE receptor 2 trafficking and cell movement.. FASEB J.

[31] Pennings MT, Derksen RH, van Lummel M, Adelmeijer J, VanHoorelbeke K (2007). Platelet adhesion to dimeric beta-glycoprotein I under conditions of flow is mediated by at least two receptors: glycoprotein Ibalpha and apolipoprotein E receptor 2′.. J Thromb Haemost.

[32] White TC, Berny MA, Tucker EI, Urbanus RT, de Groot PG (2008). Protein C supports platelet binding and activation under flow: role of glycoprotein Ib and apolipoprotein E receptor 2.. J Thromb Haemost.

[33] Urbanus RT, Pennings MT, Derksen RH, de Groot PG (2008). Platelet activation by dimeric beta2-glycoprotein I requires signaling via both glycoprotein Ibalpha and apolipoprotein E receptor 2′.. J Thromb Haemost.

[34] White-Adams TC, Berny MA, Tucker EI, Gertz JM, Gailani D (2009). Identification of coagulation factor XI as a ligand for platelet apolipoprotein E receptor 2 (ApoER2).. Arterioscler Thromb Vasc Biol.

[35] Coppinger JA, O'Connor R, Wynne K, Flanagan M, Sullivan M (2009). Moderation of the platelet releasate response by aspirin.. Blood.

[36] Yang WL, Godwin AK, Xu XX (2004). Tumor necrosis factor-alpha-induced matrix proteolytic enzyme production and basement membrane remodeling by human ovarian surface epithelial cells: molecular basis linking ovulation and cancer risk.. Cancer Res.

[37] Zhang J, Patel L, Pienta KJ (2010). Targeting Chemokine (C-C motif) Ligand 2 (CCL2) as an Example of Translation of Cancer Molecular Biology to the Clinic.. Prog Mol Biol Transl Sci.

[38] Zhang L, Blackwell K, Altaeva A, Shi Z, Habelhah H (2011). TRAF2 phosphorylation promotes NF-{kappa}B-dependent gene expression and inhibits oxidative stress-induced cell death.. Mol Biol Cell.

[39] Yanagie H, Hisa T, Ono M, Eriguchi M (2010). [Chemokine and chemokine receptor related to cancer metastasis].. Gan To Kagaku Ryoho.

[40] Chandler LA, Sosnowski BA, McDonald JR, Price JE, Aukerman SL (1998). Targeting tumor cells via EGF receptors: selective toxicity of an HBEGF-toxin fusion protein.. Int J Cancer.

[41] Mullenders J, Fabius AW, van Dongen MM, Kuiken HJ, Beijersbergen RL (2010). Interleukin-1R-associated kinase 2 is a novel modulator of the transforming growth factor beta signaling cascade.. Mol Cancer Res.

[42] Dong H, Chen L (2003). B7-H1 pathway and its role in the evasion of tumor immunity.. J Mol Med.

[43] Dong H, Strome SE, Salomao DR, Tamura H, Hirano F (2002). Tumor-associated B7-H1 promotes T-cell apoptosis: a potential mechanism of immune evasion.. Nat Med.

[44] Azuma T, Yao S, Zhu G, Flies AS, Flies SJ (2008). B7-H1 is a ubiquitous antiapoptotic receptor on cancer cells.. Blood.

[45] Miesbach W (2009). Malignancies and catastrophic anti-phospholipid syndrome.. Clin Rev Allergy Immunol.

[46] Choi SY, Gonzalvez F, Jenkins GM, Slomianny C, Chretien D (2007). Cardiolipin deficiency releases cytochrome c from the inner mitochondrial membrane and accelerates stimuli-elicited apoptosis.. Cell Death Differ.

[47] Schlumbrecht MP, Xie SS, Shipley GL, Urbauer DL, Broaddus RR (2010). Molecular clustering based on ERα and EIG121 predicts survival in high-grade serous carcinoma of the ovary/peritoneum.. Mod Pathol.

[48] Deng L, Feng J, Broaddus R (2010). The novel estrogen-induced gene EIG121 regulates autophagy and promotes cell survival under stress.. Cell Death Dis.

[49] Avgeris M, Mavridis K, Scorilas A (2010). Kallikrein-related peptidase genes as promising biomarkers for prognosis and monitoring of human malignancies.. Biol Chem.

[50] Prezas P, Arlt MJ, Viktorov P, Soosaipillai A, Holzscheiter L (2006). Overexpression of the human tissue kallikrein genes KLK4, 5, 6, and 7 increases the malignant phenotype of ovarian cancer cells.. Biol Chem.

[51] Aktas B, Tewes M, Fehm T, Hauch S, Kimmig R (2009). Stem cell and epithelial-mesenchymal transition markers are frequently overexpressed in circulating tumor cells of metastatic breast cancer patients.. Breast Cancer Res.

[52] Bonnomet A, Brysse A, Tachsidis A, Waltham M, Thompson EW (2010). Epithelial-to-mesenchymal transitions and circulating tumor cells.. J Mammary Gland Biol Neoplasia.

