# Supplementary figures and images for: Platelet Adhesion and Degranulation Induce Pro-Survival and Pro-Angiogenic Signalling in Ovarian Cancer Cells
Source: PLoS One. 2011 Oct 12;6(10):e26125. doi: 10.1371/journal.pone.0026125 (PMC3192146; doi:10.1371/journal.pone.0026125)

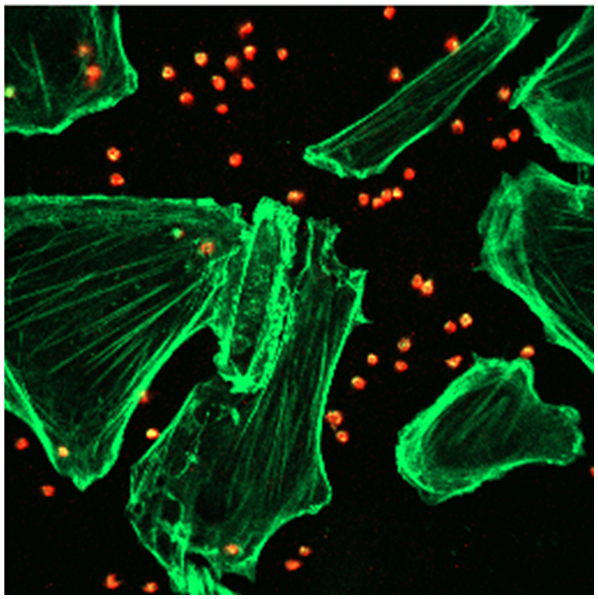

Supplement: Figure S1 — Platelet adhesion to HIO-80 cells is minimal. Fluorescence microscopy image demonstrating that platelet adhesion to HIO-80 cells is minimal. HIO-80 cells and platelets were stained for actin [green], platelets were stained specifically for CD42a [red]. (TIF) [file pone.0026125.s001.tif]
